# Glibenclamide Reduces Primary Human Monocyte Functions Against Tuberculosis Infection by Enhancing M2 Polarization

**DOI:** 10.3389/fimmu.2018.02109

**Published:** 2018-09-19

**Authors:** Chidchamai Kewcharoenwong, Satria A. Prabowo, Gregory J. Bancroft, Helen A. Fletcher, Ganjana Lertmemongkolchai

**Affiliations:** ^1^Mekong Health Science Research Institute, Khon Kaen, Thailand; ^2^Faculty of Associated Medical Sciences, The Centre for Research and Development of Medical Diagnostic Laboratories, Khon Kaen University, Khon Kaen, Thailand; ^3^Department of Immunology and Infection, Faculty of Infectious and Tropical Diseases, London School of Hygiene and Tropical Medicine, London, United Kingdom; ^4^Tuberculosis Centre, London School of Hygiene and Tropical Medicine, London, United Kingdom

**Keywords:** glibenclamide, *Mycobacterium tuberculosis*, monocyte, diabetes mellitus, M2 polarization, anti-diabetic drug

## Abstract

Tuberculosis (TB) is a global public health problem, which is caused by *Mycobacterium tuberculosis* (Mtb). Type 2 diabetes mellitus (T2DM) is one of the leading predisposing factors for development of TB after HIV/AIDS. Glibenclamide is a widely used anti-diabetic drug in low and middle-income countries where the incidence of TB is very high. In a human macrophage cell line, glibenclamide, a K^+^ATP-channel blocker, promoted alternative activation of macrophages by enhancing expression of the M2 marker CD206 during M2 polarization. M2 macrophages are considered poorly microbicidal and associated with TB susceptibility. Here, we investigated the effect of glibenclamide on M1 and M2 phenotypes of primary human monocytes and further determined whether specific drug treatment for T2DM individuals influences the antibacterial function of monocytes in response to mycobacterial infection. We found that glibenclamide significantly reduced M1 (HLA-DR^+^ and CD86^+^) surface markers and TNF-α production on primary human monocytes against mycobacterial infection. In contrast, M2 (CD163^+^ and CD206^+^) surface markers and IL-10 production were enhanced by pretreatment with glibenclamide. Additionally, reduction of bactericidal activity also occurred when primary human monocytes from T2DM individuals who were being treated with glibenclamide were infected with Mtb *in vitro*, consistent with the cytokine responses. We conclude that glibenclamide reduces M1 and promotes M2 polarization leading to impaired bactericidal ability of primary human monocytes of T2DM individuals in response to Mtb and may lead to increased susceptibility of T2DM individuals to TB and other bacterial infectious diseases.

## Introduction

Tuberculosis (TB) is a global public health problem, which is the leading cause of death due to a single infectious agent, *Mycobacterium tuberculosis* (Mtb). In 2016, TB resulted in 1.3 million deaths and 6.3 million new cases, and it is estimated that about one-quarter of the human population is latently infected (1, 2). In many tropical countries, such as Thailand, TB is an important cause of death and primarily a disease of the lung, which serves as a port of entry and a site of disease manifestation. Type 2 diabetes mellitus (T2DM) is an important risk factor for development of TB (3). A global overview focusing specifically on Asian countries with a high TB-DM burden indicates a TB prevalence 1.8–9.5 times higher among DM patients when compared to the general population (4). The predictive factors for TB among those with DM are HIV co-infection, age (older than 45), overweight, poor glycemic control, and being male (5, 6). However, this underlying immunological mechanisms are still poorly understood. Given the lack of an effective vaccine to protect adults against TB in the tropics, the problems of antibiotic resistance and the predictions that the global burden of T2DM could reach almost 600 million people in the next 20 years (7), understanding the mechanisms by which diabetes predisposes to this infection is essential. Glibenclamide rINN (glyburide USAN, sulfonylurea group) is a widely commonly used anti-diabetic drug in low and middle-income countries where the incidence of TB is high (1). The drug acts by binding to and inhibiting the ATP-sensitive potassium channel (K_ATP_) inhibitory regulatory subunit sulfonylurea receptor 1 (SUR1) in pancreatic beta cells, then increases the plasma insulin concentrations (8). This drug lowers blood glucose concentrations by about 20% and HbA1c by 1–2% (9). However, glibenclamide has the side effects such as hypoglycemia and reduced immune functions through inhibition of inflammasome (8) and Atp binding cassette transporter (10). Our previous study showed that glibenclamide has potent and wide-ranging effects on cell mediated immune responses including reduced neutrophil pro-inflammatory cytokine production, migration, and killing in response to another intracellular bacteria, Burkholderia pseudomallei (11, 12).

Monocytes and macrophages are the primary target of Mtb, and their innate capacity to control Mtb defines the early progression of the infection (13). In peripheral blood, monocyte numbers expand during active TB disease (14). *In vitro* study on diabetic cells found reduced level of Mtb phagocytosis possibly due to alteration in diabetic monocytes and complement system (15). Monocytes can differentiate into M1 or M2 macrophages with pro- or anti-inflammatory functional phenotypes, respectively (16). An M1 phenotype is associated with the up-regulation of MHC-II molecules (such as HLA-DR) (17) and a co-stimulatory receptor, CD86 and the ability to produce pro-inflammatory cytokines such as TNF-α and IL-1β (16, 18, 19). Alternatively, the M2 phenotype can be characterized by the upregulation of the scavenger receptors, CD163 and the mannose receptor, CD206, as well as the ability to release anti-inflammatory cytokines, such as IL-10 (16, 20). Generally, M1 macrophages are considered part of the common host response against intracellular bacteria and involved in killing of mycobacteria, while M2 macrophages are associated with tissue repair and bacterial persistence (13, 21). The polarization state of monocytes is likely important for maintenance of a balanced inflammatory response in TB disease. In a human macrophage cell line, glibenclamide promoted alternative activation of macrophages by enhancing the expression of the M2 marker CD206 during M2 polarization (22). However, to date, there is no information as to how glibenclamide affects primary human monocyte phenotype and function in response to mycobacterial infection. Here, we demonstrated the effect of glibanclamide on M1 and M2 phenotypes of primary human monocytes against BCG and Mtb *in vitro* and also investigated whether drug treatment for T2DM individuals influences cytokine production and killing activity by monocytes in response to mycobacterial infection. We conclude that glibenclamide reduces M1 markers and enhances M2 markers on primary human monocytes, which leads to reduced killing activity against Mtb. Our findings suggest that treatment with glibenclamide impairs the anti-bacterial defense functions of human monocytes in DM individuals.

## Materials and methods

### Participants

We collected whole blood from 10 healthy individuals at LSHTM, UK and 41 diabetic, and 15 healthy control Thai individuals enrolled at Yang Lum Health Promoting Hospital, Ubon Ratchathani, Thailand. All individuals had no signs of acute infectious disease in the 3 months prior to enrollment. We classified diabetic individuals according to drug treatment, divided into three groups: (1) glibenclamide alone or both glibenclamide and metformin, (2) glipizide alone, and (3) metformin alone. Diabetic individuals from each group exhibited impaired glycemic control based on HbA1c levels (>6.5%). Exclusion criteria for both healthy and diabetic volunteers included impaired renal function, defined by a serum creatinine level of ≥2.2 mg/dl.

### Ethics statement

This study was carried out in accordance with the recommendations of UK and Thailand guidelines for human research and the protocol was approved by LSHTM Research Ethics Committee and Nakhon Phanom Hospital Ethical Review Committee for Human Research. All subjects gave written informed consent in accordance with the Declaration of Helsinki.

### Microorganisms

Stocks of Mtb H37Rv or Mycobacterium bovis Bacille Calmette-Guerin (BCG) Pasteur-Aeras were cultured in 7H9-OADC-Tween-Glycerol for 14 days. Bacterial growth was assessed by measuring the optical density at 600 nm and the number of viable bacteria (colony-forming units) in inocula determined by retrospective plating of serial ten-fold dilutions on 7H11 agar, and then frozen at −80°C. Live Mtb was handled under Advisory Committee on Dangerous Pathogens (UK) bio-containment level 3 conditions at LSHTM and Khon Kaen University.

### Monocyte isolation

We isolated human peripheral blood mononuclear cells (PBMCs) from heparinized venous blood by Ficoll-Paque centrifugation. PBMC suspensions at 10^7^ cells/ml were plated 300 μl in each respective well of a 48-well plate and incubated for 2 h. The non-adherent cells were removed by repeated pipetting and washed with 10% FBS in RPMI 1640 culture medium for three times. Fresh medium was added to the adherent cells. The resulting cell preparation was confirmed to consist of >95% monocytes by Giemsa staining and microscopy.

### Monocyte stimulation and cytokine measurement

Unless stated otherwise, purified monocytes at a concentration 2.5 × 10^5^ cells/ml in RPMI 1640 culture medium were pretreated with 50 μM glibenclamide (Sigma) [comparable to the peak human plasma concentration achieved following a 20 mg oral dose (23)] for 30 min and then infected with live Mtb or BCG at 10^2^ or 10^5^ CFU per well or activated with 10 μg/ml of LPS (from Escherichia coli, Sigma) at 37°C for 96 h. The supernatants were stored at −80°C until cytokines were measured. TNF-α, IL-10, and IL-6 concentrations were tested in duplicate by ELISA (Invitrogen and BD Biosciences) according to the manufacturer's instructions. IL-1β concentration was measured using Quantikine HS ELISA (R and D system). IL-8, MCP-1, RANTES, IP-10, and MIG were determined using a cytometric bead array multiplex assay (CBA) in accordance with the manufacturer's instructions (BD Biosciences). All cytokine data in response to Mtb, BCG, or LPS were subtracted from the medium control of each sample.

### Cell surface marker staining

Following incubations, the plate was incubated in 4°C for 30 min and rubbed gently by pipette. Then, suspended monocytes were collected and transferred to FACS tubes. Then, cells were centrifuged and washed with 1 ml FACS buffer. Pelleted cells were surface stained with anti-CD14-BV421, anti-CD16-BV510, anti-CD86-PE-Cy7, anti-HLA-DR-PE, anti-CD206-APC, and anti-CD163-BV605 (BioLegend) for 30 min at 4°C. After washing with FACS buffer, cells were fixed by 4% paraformaldehyde (Sigma, UK) for 10 min at 4°C and then washed by FACS buffer. Finally, cells were resuspended in 250 μl FACS buffer and kept in 4°C until analysis. Data was acquired using an LSRII flow cytometer (BD Biosciences) configured with three lasers and 10 detectors and FACSDiva acquisition software (BD Biosciences). Compensation was performed using tubes of CompBeads (BD Biosciences) individually stained with each fluorophore and compensation matrices were calculated with Flowjo version 10 (TreeStar Inc., Ashland, OR, USA).

### *In vitro* mycobacterial growth inhibition assay (MGIA)

Purified monocytes were pretreated with or without glibenclamide for 30 min as described and then infected with live Mtb or live BCG at 10^2^ CFU for 96 h with glibenclamide in the condition. Following incubations, cells and remaining Mtb or BCG were pelleted and cells were lysed by incubation in sterile water with vortexing three times in between. Mtb or BCG from each individual tube were then transferred into a corresponding MGIT tube and time to positivity was determined using a MGIT 960 (Becton Dickinson). Direct-to-MGIT controls were used for the calculation of relative growth. All mycobacteria growth inhibition assays were carried out in duplicate. For each tube, time to positivity in hours was converted to log CFU of bacteria using a previously determined standard curve for the stock of Mtb or BCG used (24).

### Statistics

Statistical analysis (One way ANOVA and paired t-test) was performed by using Graphpad PRISM statistical software (Graphpad). P-values ≤ 0.05 were considered significant. The statistical power of the study was calculated by *post-hoc* power analysis for all experiments measuring cytokine production in diabetic individuals and there we have >80% power with 95% confidence to detect differences between groups.

## Results

### Glibenclamide reduces M1 while enhancing M2 surface marker expression on primary human monocytes

**Figure 1:**
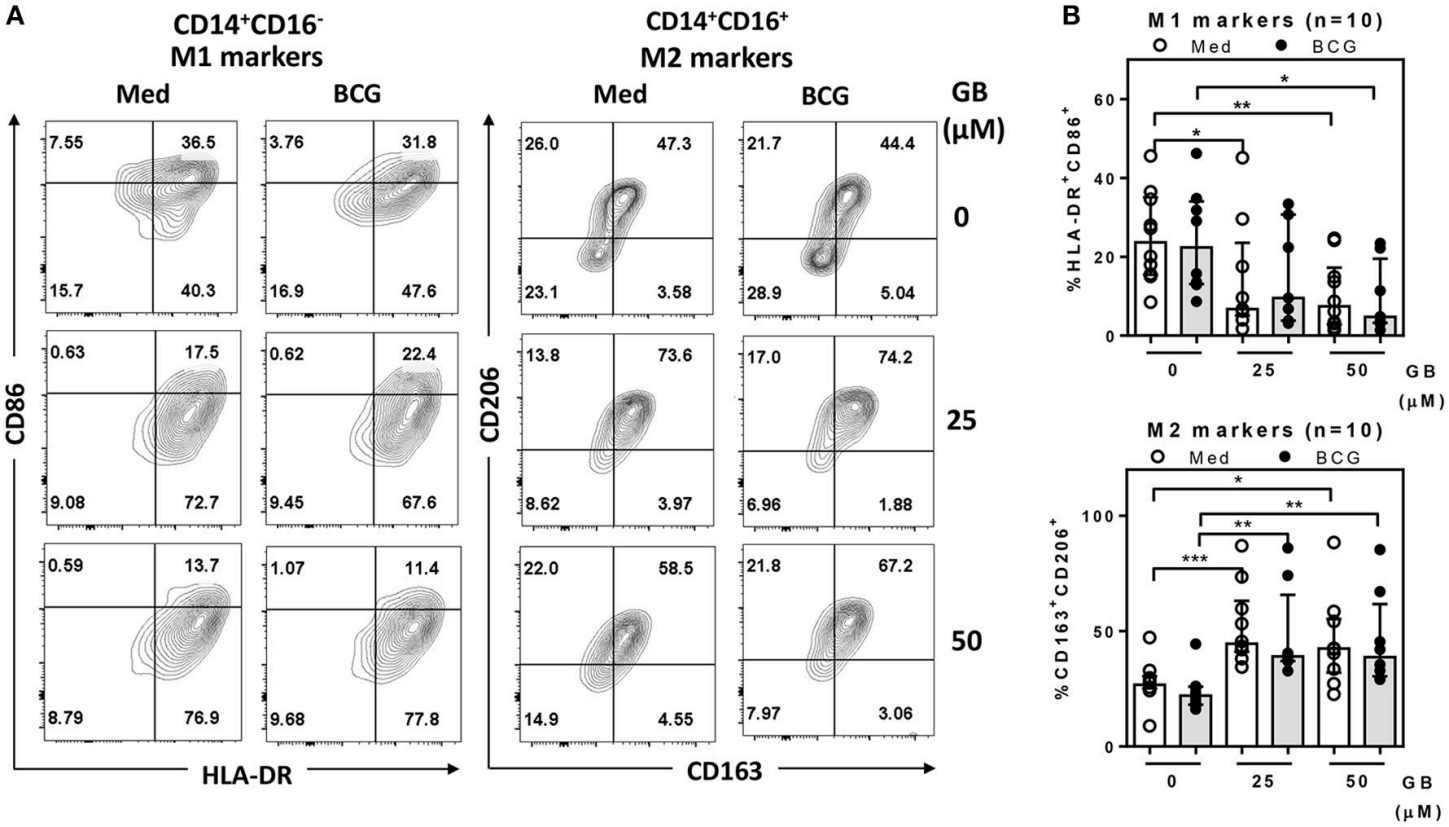
Glibenclamide reduces M1 but enhances M2 markers on human monocytes. Monocytes from UK healthy individuals (n = 10) were treated with glibenclamide [GB, 0 (vehicle, DMSO), 25, 50 μM] for 30 min. Drug-treated monocytes were incubated with 100 CFU of BCG or RPMI medium (Med) for 96 h and then analyzed M1 and M2 markers by flow cytometry. **(A)** After exclusion of debris and doublets, monocytes were detected as CD14^+^CD16^−^ and CD14^+^CD16^+^ populations and then further analyzed for M1 (HLA-DR^+^ and CD86^+^) and M2 (CD163^+^ and CD206^+^) expression, respectively. These data are representative of a healthy individual. **(B)** The percentage of M1 or M2 positive cells for each individual are shown. Each bar represents the median of each group and each dot represents the value of each sample. Statistical analysis was performed using One Way ANOVA to compare all groups, ***P < 0.001, **P < 0.01, *P < 0.05. No asterisk, non-significant.

Because the peripheral lipid portion of the cell wall is very similar between BCG and Mtb (25), it is predicted that their ability to infect peripheral monocytes or macrophages is similar. Firstly, to determine the effect of glibenclamide on M1 and M2 marker expression, purified primary monocytes from healthy control individuals were pretreated with the drug at doses comparable to the range of glibenclamide given during oral therapy to human patients (26, 27), prior to infection with BCG. In this study, we refer only to two major subsets, terming classical monocytes simply as CD14^+^CD16^−^, and non-classical as CD14^+^CD16^+^ (28). Cultured monocytes were analyzed with M1 (HLA-DR^+^ and CD86^+^) and M2 (CD163^+^ and CD206^+^) surface markers on CD14^+^CD16^−^ and CD14^+^CD16^+^, respectively (Figure 1A and Figure S1). Here, we found that glibenclamide significantly reduced M1 surface markers on CD14^+^CD16^−^ and enhanced M2 surface markers on CD14^+^CD16^+^ with or without BCG infection (Figure 1), regardless of the concentration of BCG (Figure S2). M2 surface markers were not detected on CD14^+^CD16^−^ and no difference was found with glibenclamide-pretreatment of M2 and M1 surface markers on CD14^+^CD16^−^ and CD14^+^CD16^+^ monocytes, respectively (Figure S3). To determine whether glibenclamide alters M1 and M2 surface markers in an M2 macrophage polarization model, we activated primary human monocytes with IL-4 to obtain M2 phenotype cells in the presence or absence of glibenclamide (Figure S4). With glibenclamide, M1 surface markers of CD14^+^CD16^−^ cells were significantly reduced, while an upward trend was observed in the expression of M2 surface markers (Figure S4B). Gibenclamide also clearly enhanced M2 surface marker expression (CD206) in CD14^+^CD16^−^ cells during M2 polarization (Figure S4A), consistent with the published data on human macrophage cell lines (22). We also performed an M1 macrophage polarization model, but no significant difference was found (data not shown). Nevertheless, we provide evidence that glibenclamide reduces M1 and increases M2 surface marker expression on primary human monocytes.

### Glibenclamide reduces TNF-α while enhancing IL-10 production from primary human monocytes in response to BCG and *M. tuberculosis*

**Figure 2:**
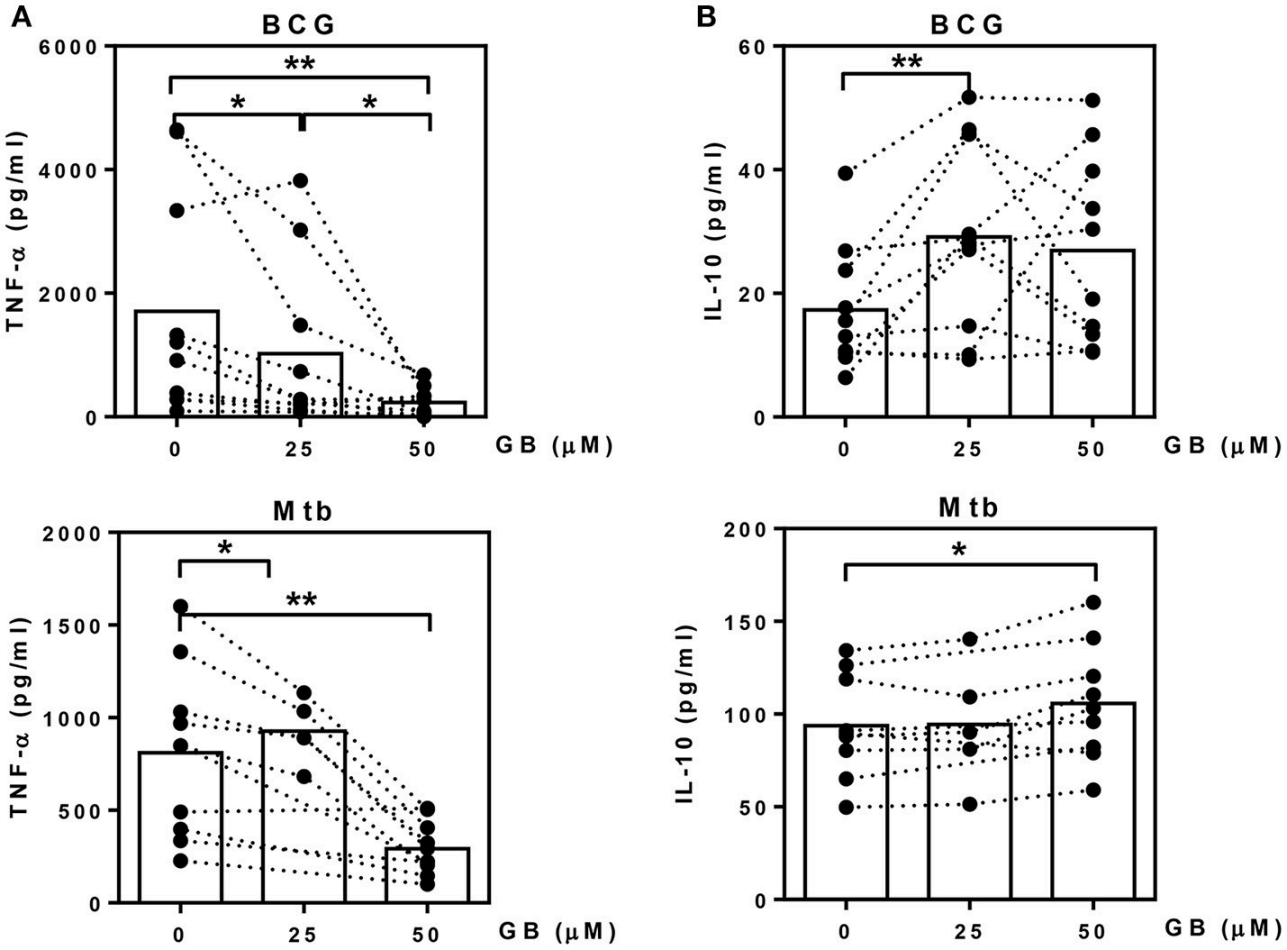
Glibenclamide reduces TNF-α while enhances IL-10 production from monocytes in response to BCG and *M. tuberculosis*. Monocytes from UK healthy individuals (n = 10) were treated with glibenclamide (GB, 25, 50 μM) for 30 min., incubated with 10^5^ CFU per well of BCG; or monocytes from UK (n = 5) and Thai (n = 5) healthy individuals were treated with glibenclamide and incubated with *M. tuberculosis* at 10^5^ CFU per well (Mtb). After incubation for 96 h, supernatants were collected for **(A)** TNF-α and **(B)** IL-10 detection. Statistical analysis was performed using One Way ANOVA or paired t-test for BCG or Mtb infected samples, respectively. Each bar is expressed as median of each group and each dot represents each sample. **P < 0.01, *P < 0.05. No asterisk, non-significant.

We next evaluated whether glibenclamide alters the ability of primary human monocytes to release pro- and anti-inflammatory cytokines in response to BCG and Mtb infection by detecting TNF-α (M1 phenotype) and IL-10 (M2 phenotype), respectively. Purified primary monocytes from healthy individuals were pretreated with glibenclamide, infected with BCG or Mtb for 96 h and cytokine concentrations measured in supernatants. Consistent with its impact on macrophage polarization, glibenclamide significantly reduced TNF-α in a concentration-dependent manner (Figure 2A). Secretion of IL-1β was significantly reduced when cells were pretreated with glibenclamide (Figure S5). In contrast, glibenclamide significantly enhanced IL-10 production in response to both BCG and Mtb from the same cell cultures (Figure 2B). Together, we conclude that glibenclamide reduces the expression of cytokines associated with M1 phenotype and enhances expression of M2 associated cytokines in primary human monocytes in response to mycobacterial infection.

### Broad cytokine production in response to *M. tuberculosis* is associated with the choice of drug treatment in individuals with diabetes mellitus

**Figure 3:**
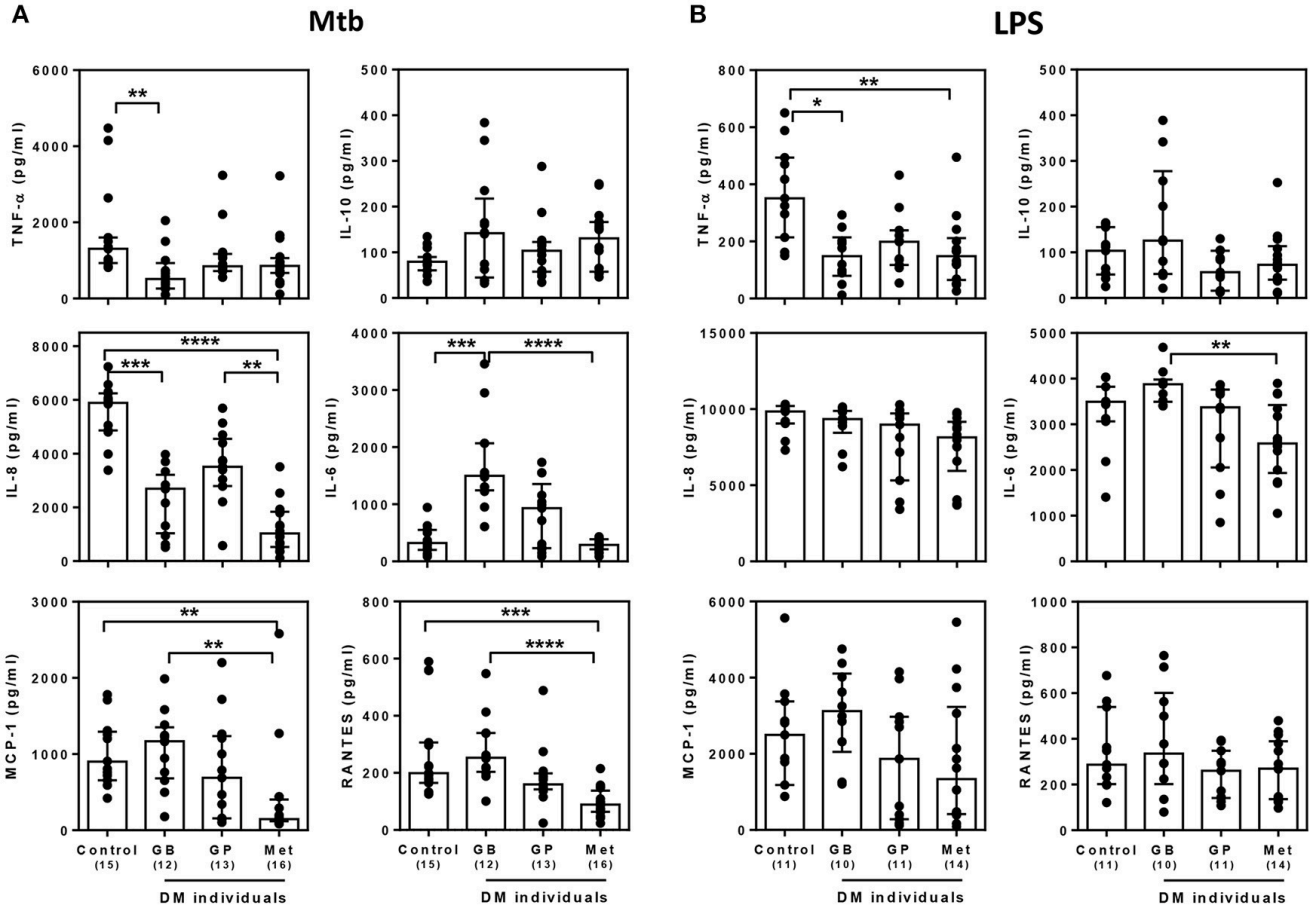
Effect of different DM treatment regimens on broad cytokine production in response to *M. tuberculosis*. Four Thai groups are shown including healthy controls and diabetic individuals who have been treated with glibenclamide (GB), glipizide (GP), or metformin (Met). Purified monocytes from each group were infected with **(A)** *M. tuberculosis* at 10^5^ CFU per well (Mtb) or stimulated with **(B)** 10 μg/ml of E. coli LPS. Due to limited blood volume, some samples were not stimulated with LPS. After incubated at 96 h, supernatants were collected and kept in −80°C prior to cytokine detection. TNF-α, IL-10, and IL-6 were detected by ELISA and IL-8, MCP-1 and RANTES were detected by CBA. Each bar is expressed as median with interquartile range of each group and each dot represents each sample. The number of individuals tested are shown in parentheses. Asterisks indicate significant differences between all individual groups by One Way ANOVA. ****P < 0.0001, ***P < 0.001, **P < 0.01, *P < 0.05. No asterisk, non-significant.

Currently, not only glibenclamide and metformin but glipizide, a partial potassium channel blocker, is also one of the main drugs being used to control blood glucose levels in TB endemic areas (29). To investigate whether different drugs involved in the management of T2DM, vary in their effects on innate immune function, we compared the broad cytokine production including TNF-α, IL-10, IL-8, IL-6, MCP-1, RANTES, IP-10, and MIG of monocytes purified from T2DM individuals under different drug regimens (see Table 1 for characteristic of individuals). These T2DM individuals had similar levels of BMI, fasting blood glucose and markers of glycemic control (HbA1c), regardless of anti-diabetic drug treatment used (Table 1). We found that T2DM individuals who were being treated with glibenclamide had significantly lower TNF-α and IL-8 but increased IL-6 when compared to monocytes from healthy control groups in response to Mtb infection (Figure 3A). A similar effect of glibenclamide was observed upon LPS stimulation of cells, with lower levels of TNF-α produced from glibenclamide treated cells (Figure 3B). However, we only observed a trend toward increased IL-10 from monocytes from T2DM individuals who were being treated with glibenclamide against Mtb infection with or without LPS activation (Figures 3A,**B**). Moreover, T2DM individuals who were being treated with metformin had significantly reduced IL-8, IL-6, MCP-1, and RANTES in the presence of Mtb (Figure 3A) and reduced TNF-α and IL-6 in response to LPS (Figure 3B). IP-10 and MIG levels were lower than the limit of detection in this experiment. Our data suggests that in T2DM individuals, glibenclamide reduces an M1 phenotype, especially TNF-α and IL-8 production in primary human monocytes in response to Mtb and LPS. Moreover, metformin reduces IL-6 and chemokines which are involved with macrophage polarization.

**Table 1:** General characteristics of diabetic and healthy control individuals.

	**Diabetic (n** = **41)**			
**Individual groups**	**Healthy**	**Glibenclamide^c^**	**Glipizide**	**Metformin**
Total (n = 56)	15	12	13	16
Sex (female: male)	13:2	5:7	8:5	11:5
Average age (year)^a^	47 ± 7	59 ± 9^b^	64 ± 10^b^	61 ± 10^b^
BMI (kg/m^2^)^a^	24.5 ± 3.1	24.5 ± 2.6^b^	24.6 ± 2.4^b^	24.4 ± 2.6^b^
Fasting blood sugar (mg%)^a^	ND	169.9 ± 52.4^b^	141.7 ± 27.2^b^	145.0 ± 37.2^b^
HbA1c (%)^a^	5.3 ± 0.4	8.4 ± 2.3^b^	7.8 ± 1.9^b^	7.5 ± 2.1^b^

*ND, not determined*.

a *The values are means ± SD*.

b *No statistically significant differences (P ≥ 0.05) compared across all diabetic individuals using One Way ANOVA*.

c *Glibenclamide alone, n = 6 and combination with metformin, n = 6*.

### Glibenclamide treatment impairs killing of *M. tuberculosis* by primary human monocytes

**Figure 4:**
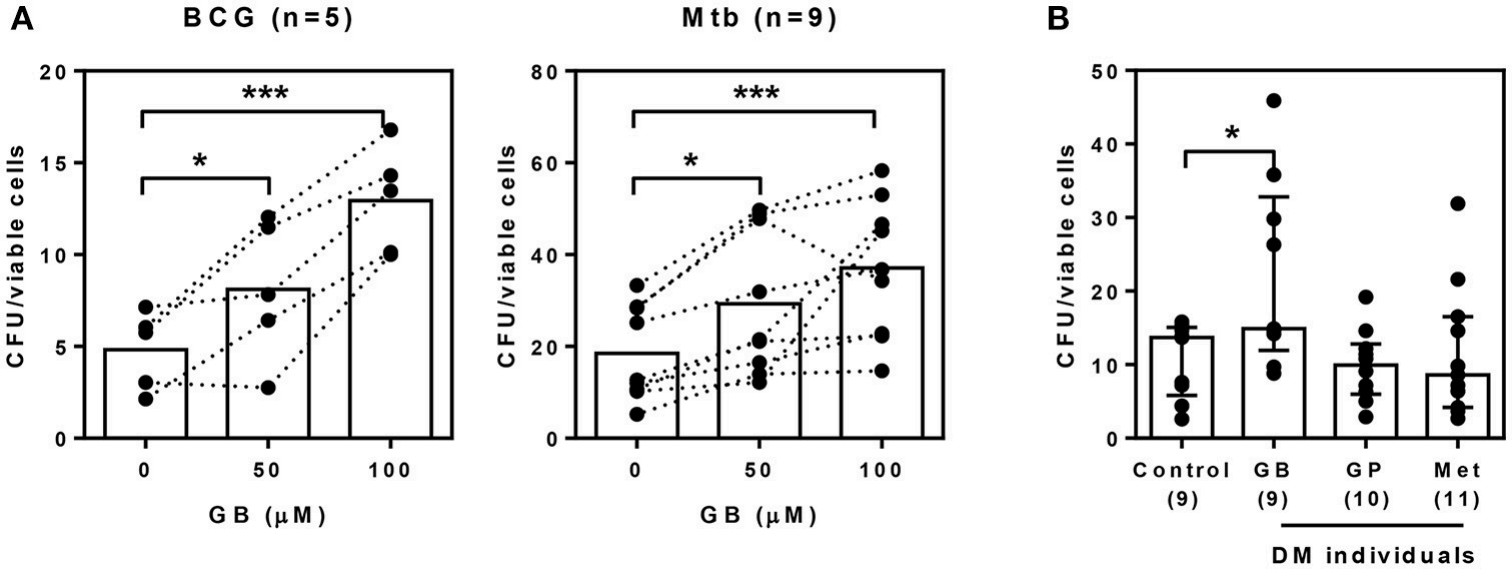
Glibenclamide treatment impairs killing of *M. tuberculosis* by monocytes. **(A)** Monocytes from UK healthy individuals were treated with glibenclamide (GB, 50, 100 μM) for 30 min. Drug-treated monocytes were incubated with 10^2^ CFU per well of *M. tuberculosis* (Mtb, n = 9) or BCG (n = 5) for 96 h and then the total bacteria were collected for MGIA. **(B)** Purified monocytes from four Thai individual groups shown as healthy control (n = 9) and diabetic individuals who have been treated with glibenclamide (GB, n = 9), glipizide (GP, n = 10), or metformin (Met, n = 11) were infected with 10^2^ CFU of *M. tuberculosis* for 96 h and total bacteria were determined by MGIA. Statistical analysis was performed using One Way ANOVA to compare between control and other groups. Data are expressed as median with interquartile range. ***P < 0.001, * *P* < 0.05. No asterisk, non-significant.

Our previous data suggested that glibenclamide promotes an M2 phenotype in T2DM individuals. Moreover, in other studies, the shift of polarization toward M2 is associated with poor microbicidal activity and parallels with TB susceptibility (21). We further investigated the effect of glibenclamide on the killing function of primary human monocytes using a mycobacterial growth inhibition assay (MGIA) (24). Bacterial growth, which was measured after culture with primary monocytes for 96 h, showed that glibenclamide significantly reduced the ability of monocytes to eliminate Mtb and also BCG in a concentration-dependent manner (Figure 4A). Furthermore, to examine whether specific drug treatment for T2DM individuals influenced the antimicrobial functions of monocytes, purified monocytes from either healthy or T2DM individuals (see Table 1 for details of individuals) were exposed to Mtb and the bacterial growth was assessed by MGIA. Monocytes of T2DM individuals who were being treated with glibenclamide had impaired killing activity compared to monocytes from healthy controls as well as other anti-diabetic drug treatment groups (Figure 4B). These data implied that glibenclamide impairs the antimycobacterial function of primary human monocytes.

## Discussion

Individuals with T2DM have an increased risk of developing infections and sepsis (30, 31). Previous studies show that phagocyte function is compromised (32) and that antioxidant systems and adaptive immunity may be depressed in individuals with T2DM (31). Many conditions are strongly associated with T2DM, including malignant otitis externa, emphysematous pyelonephritis, emphysematous cholecystitis, Klebsiella liver abscesses, rhinocerebral mucormycosis (33), urinary tract infection by E. coli (34), salmonellosis (35), TB (3), and melioidosis (36). Our previous studies show that not only T2DM physiology itself, but also anti-diabetic drug treatment reduced neutrophil functions of diabetic individuals in response to B. pseudomallei infection, which caused melioidosis (11, 12). These Mtb and B. pseudomallei infections share many features including the importance of cell mediated immunity for immune defense, generation of granulomatous pathology in infected tissues, prolonged periods of clinical latency, an interferon dominant host transcriptional profile and difficulty in generating sterilizing immunity (36–38). This is of particular relevance to increased risk of TB in individuals with T2DM, yet the understanding of the immunological changes, which underlie this susceptibility are still not defined.

In this study, we focus on the possible impact of anti-diabetic drugs on monocytes from diabetic individuals against mycobacterial infection as monocytes are key mediators of Mtb infection and resistance (13). Many studies indicate that human monocytes subsets respond differentially to Mtb infection (39–43). CD14^+^CD16^+^ monocytes have recently been shown to support Mtb replication as and there is a correlation between the abundance of CD14^+^CD16^+^ cells and the progression of TB disease (13, 39, 40). Although, binding and ingestion of microorganisms during non-opsonic phagocytosis had been reported through the mannose receptor, CD206 (43), our data showed that glibenclamide enhances CD206 (M2 marker) on CD14^+^CD16^+^ monocytes and this was associated with a reduction in mycobacterial killing. At the transcriptome level, M2 macrophages displayed a diminished inflammatory response to Mtb as reflected by reduced nitric oxide (NO) production and increased iron availability, suggesting these monocytes offer a permissible intracellular environment for bacterial replication (44). Moreover, our data also showed that glibenclamide reduced MHC-II molecules, HLA-DR, and a co-stimulatory receptor, CD86 which are involved in antigen presentation and T cell co-stimulation (21), implying that monocytes treated with glibenclamide are less efficient in triggering T cell responses compared to non-treated monocytes. These data are consistent with a study in HIV negative TB patients with T2DM in Tanzania. They found that hyperglycemia was inversely correlated with live BCG-specific CD4^+^ T cell responses in patients with latent or active TB and that half of these diabetic patients were prescribed with glibenclamide alone in combination with other anti-diabetic drugs (45). On the other hand, the novel monosubstituted sulfonylureas could inhibit Mtb replication of both H37Rv and extensively drug-resistant strains in lungs of mice through targeting acetohydroxyacid synthase (46). This latter study suggests that modified sulfonylureas may be effective as potential drug candidates against TB.

Since our previous data in human neutrophils from diabetic individuals who have been treated with glibenclamide alone and in combination with metformin showed a similar cytokine pattern against B. pseudomallei infection (11), and the majority of diabetic individuals who have been treated with sulfonylureas are also treated with metformin [as recommended by the American Diabetes Association's (47)], data from those diabetic individuals treated with glibenclamide alone or in combination were combined for immune analysis, unless stated otherwise. TNF-α is a major cytokine of the M1 pathway (14) and depletion of TNF causes a relative increase in M2 gene expression, thereby favoring the M2 pathway (exemplified by the presence of IL-14 or IL-13) (48, 49). In an Mtb infection model, TNF-α depletion resulted in increased susceptibility, with mice succumbing to infection within 2–3 weeks, while harboring a high bacterial burden (50). Also, chemokines such as IL-8 (CXCL8), MCP-1 (CCL2), and RANTES (CCL5) are produced at high levels in M1 macrophages (51). In this study, we not only observed a reduction of TNF-α but also IL-8 production from monocytes of T2DM individuals who were being treated with glibenclamide. Surprisingly, we also found that IL-6 was significantly enhanced in monocytes of the glibenclamide treatment group. IL-6 exerts a pro-inflammatory (52) or an anti-inflammatory (53) effect dependent on the local immune microenvironment. IL-6 can induce M2 macrophage differentiation through STAT3 activation and can enhance infiltration of CD163+CD206+ macrophages in gastric tumor tissue (54). Moreover, IL-6 production by Mtb-infected macrophages inhibited uninfected macrophage responses to IFN-γ (55). These previous reports support our data significantly showing that T2DM individuals who were being treated with glibenclamide have reduced TNF-α and IL-8 while it enhances IL-6 production and M2 surface markers on primary human monocytes, leading to impair mycobacterial killing (even though some of DM individuals who were being treated with glibenclamide were also being treated with metformin in combination).

In contrast, IL-10 is a hallmark M2 cytokine in both mice and human (21). However, we only observed a trend of IL-10 increase in T2DM individuals who have been treated with glibenclamide. The reason that we could not clearly see a significant enhancement of IL-10 could be due to (1) length of culture as IL-10 might be used by monocytes after 96 h culture, and (2) IL-10 is a potent anti-inflammatory cytokine that plays a crucial, and often essential, role in preventing inflammatory pathology and it is produced as a synthesis inhibitory factor for a negative feedback mechanism to limit over pro-inflammatory cytokine response toward Mtb infection (50). Once pro-inflammatory cytokines have been suppressed, IL-10 might not need to be plentifully produced. Nevertheless, we have shown that glibenclamide enhances IL-10 levels *in vitro*. In Thailand, East Asian (w/Beijing) strains predominated in both TB meningitis and pulmonary TB disease (56). However, our study used only Mtb H37Rv, the most studied strain of TB in research laboratories (57), and BCG, with which most Thai people have been vaccinated (58). It is possible that the magnitude of cytokines produced from human monocytes may be different in response to East Asian Mtb strains.

Obesity and T2DM are now recognized as chronic proinflammatory diseases (59). Previous studies found that short-chain fatty acids inhibit Mtb-induced pro-inflammatory cytokine production from human PBMCs (60), and poor glycemic control is a risk factor for TB infection (61). Moreover, an imbalance in the ratio of M1 and M2 macrophages, with increased cytokine production from M1 macrophages and/or reduced anti-inflammatory signals from M2 macrophages leads to adipose tissue dysfunction and impairs glucose tolerance. However, the characteristics of our samples showed that Thai DM individuals had similar BMI results as healthy controls at the time of enrollment. Also, previous studies proposed that M2 macrophages strongly promote pancreatic beta-cell proliferation (62), with enhancing beta-cell mass could be an ideal cure for DM. Linking these observations to our data, indicating that glibenclamide promotes M2 markers, suggests that another positive effect of glibenclamide on diabetes is carried out by macrophages exhibiting an M2 phenotype. The K_ATP_ channel is also known to influence the phenotype of prepolarized macrophages and inhibition of K_ATP_ channel promotes M2, while opening of K_ATP_ channel augments M1 marker expression in a human monocyte cell line (22). Therefore, as glipizide is a partial inhibitor of K_ATP_ channel, we could not expect to observe an effect on cytokine production and killing activity of primary human monocytes against Mtb infection. Another major anti-diabetic drug, metformin, a candidate for host-directed therapy for TB (63), was reported to reduce pro-inflammatory cytokine production in response to E. coli LPS (64) and approach to target Mtb by pharmacologically stimulating intracellular mycobacteria clearance through autophagy (65). Moreover, metformin was observed to inhibit macrophage differentiation via AMPK-mediated inhibition of STAT3 activation and to inhibit TNF-α and MCP-1 production (66). These are consistent with our data, which show a reduction in TNF-α and IL-6 against LPS and IL-6, IL-8, MCP-1, and RANTES in response to Mtb infection in T2DM individuals who were being treated with metformin. However, the killing function of monocytes from T2DM who were being treated with metformin is not impaired.

The possible mechanism to explain how glibenclamide is associated with M1 and M2 marker alteration could be (1) pre-differentiated/pre-polarized macrophages presented an expression pattern of potassium subunits that facilitated more efficient glibenclamide binding (22) and might directly modulate macrophage polarization, and (2) the reduction of IL-1β level through inflammasome which triggered the inhibition of potassium channel by glibenclamide (8) might result in cytokine imbalance, especially TNF-α, IL-8, and IL-6 in this study and lead to M1 and M2 marker alteration. This switch between M1 and M2 state may indicate how the innate immune balance is maintained by macrophage subsets during bacterial infection.

Taken together, this is the first report to describe that glibenclamide impairs mycobactericidal ability of primary human monocytes of T2DM individuals in response to Mtb by reducing M1 and promoting M2 polarization. Our data suggests that treatment with glibenclamide may result in increased susceptibility of T2DM individuals to TB and other bacterial infectious diseases.

## Author contributions

GB, HF, and GL conceived the research, oversaw the study, and the data analysis. CK and SP performed the experiments and the data analysis. CK, SP, GB, HF, and GL interpreted the data and wrote the manuscript. All authors read, commented on, and agreed on the content of the manuscript.

### Conflict of interest statement

The authors declare that the research was conducted in the absence of any commercial or financial relationships that could be construed as a potential conflict of interest. The reviewer AT and handling Editor declared their shared affiliation.
