# Supplementary material for: Glibenclamide Reduces Primary Human Monocyte Functions Against Tuberculosis Infection by Enhancing M2 Polarization
Source: Front Immunol. 2018 Sep 19;9:2109. doi: 10.3389/fimmu.2018.02109 (PMC6157405; doi:10.3389/fimmu.2018.02109)
Supplement: Supplementary file 1 [file Data_Sheet_1.PDF]

Supporting Information

Glibenclamide reduces primary human monocyte functions against tuberculosis infection by enhancing M2 polarization

Chidchamai Kewcharoenwong[1,2], Satria A. Prabowo[3,4], Gregory J. Bancroft[3,4], Helen A. Fletcher[3,4*] and Ganjana Lertmemongkolchai[1,2*]

[1]Mekong Health Science Research Institute and [2]Centre for Research and Development of Medical Diagnostic Laboratories, Faculty of Associated Medical Sciences, Khon Kaen University, Thailand, [3]Department of Immunology and Infection, Faculty of Infectious and Tropical Diseases, London School of Hygiene and Tropical Medicine, UK, [4]Tuberculosis Centre, London School of Hygiene and Tropical Medicine, UK.

*Corresponding authors

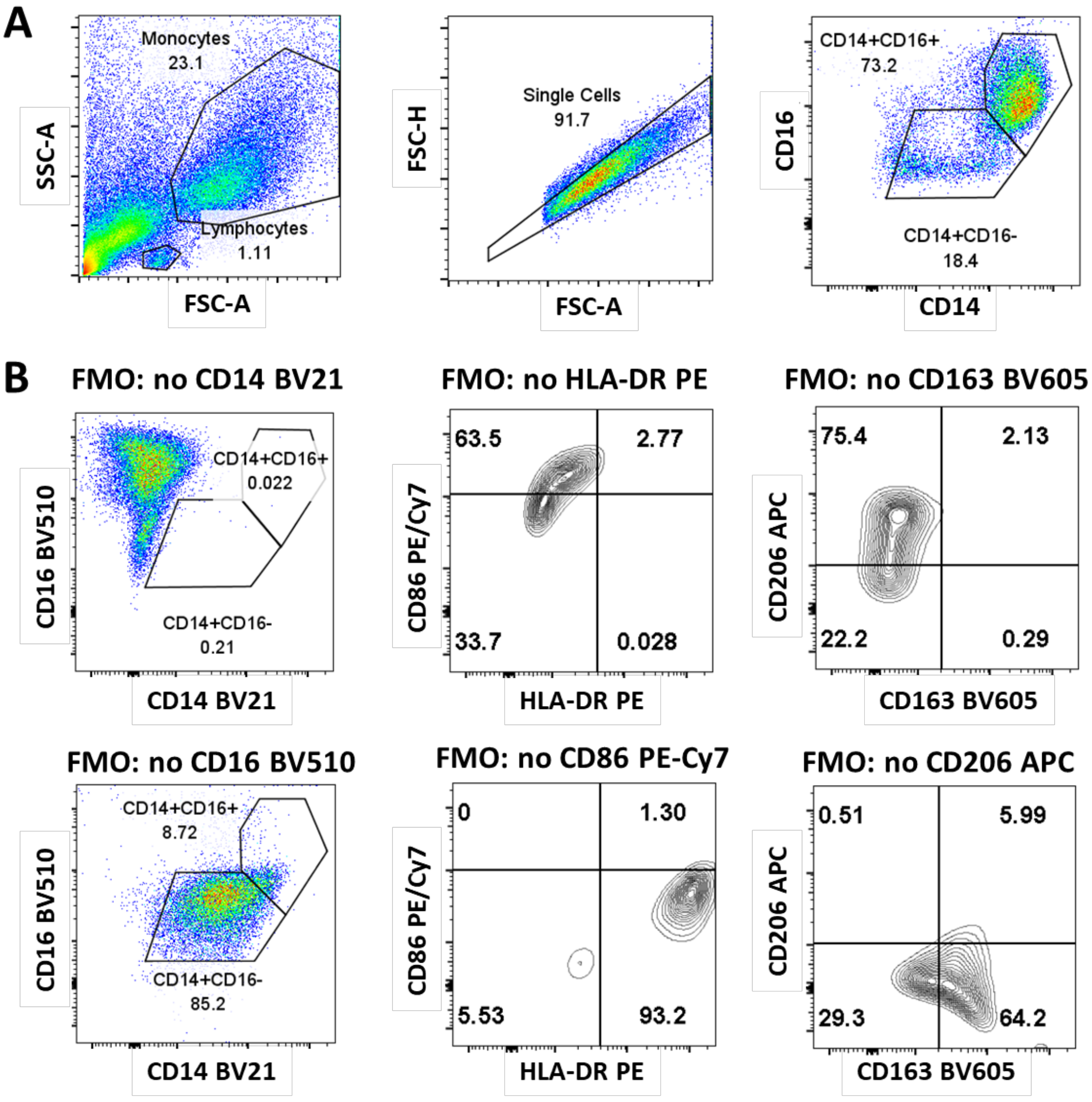

Figure S1: Gating strategy and Fluorescence minus one (FMO) controls.

(A) Total monocyte population from UK healthy individual was gated and then detected as $CD14^{+}CD16^{-}$ and $CD14^{hi}CD16^{+}$ populations. Each population was further analyzed for M1, HLA-$DR^{+}CD86^{+}$ or M2, $CD163^{+}CD206^{+}$ expression. (B) 5% contour plots with outliers show how a FMO control (left panel) was used to identify stained cells in the fully stained sample (Fig 1A). There were CD14 BV21, CD16 BV510, HLA-DR PE, CD86 PE-Cy7, CD163 BV605 and CD206 APC FMO.

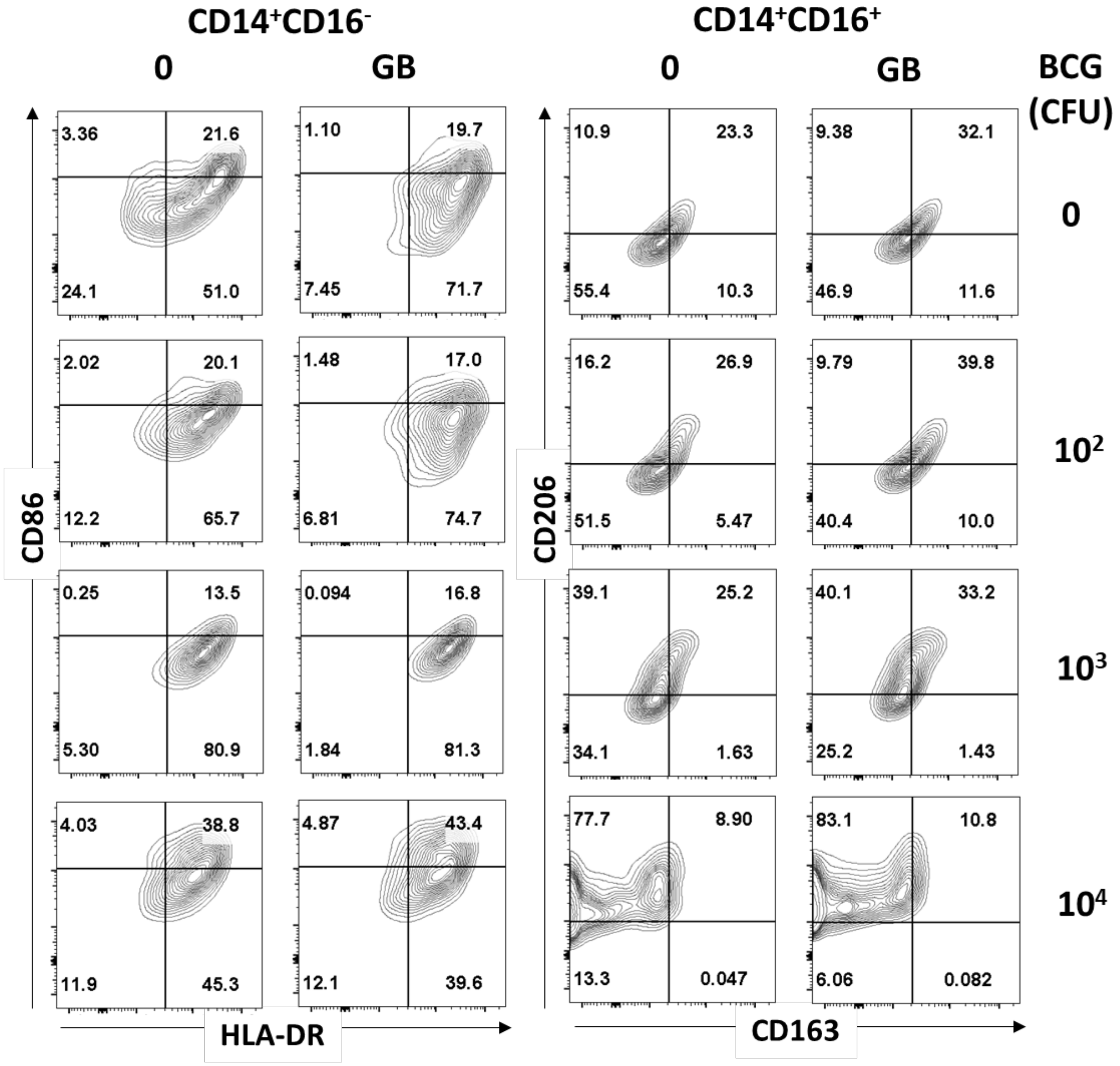

Figure S2: M1 and M2 marker expression in response to varied concentrations of BCG. Monocytes from UK healthy individuals (n=10) were treated with glibenclamide (GB, 25 μM) or vehicle (0) for 30 min., incubated with 10^2, 10^3 or 10^4 CFU of BCG or RPMI medium (Med) for 96 h and then analysed for M1 (HLA-DR$^+$CD86$^{+)}$ or M2 (CD163$^+$CD206$^{+)}$ expression by flow cytometry.

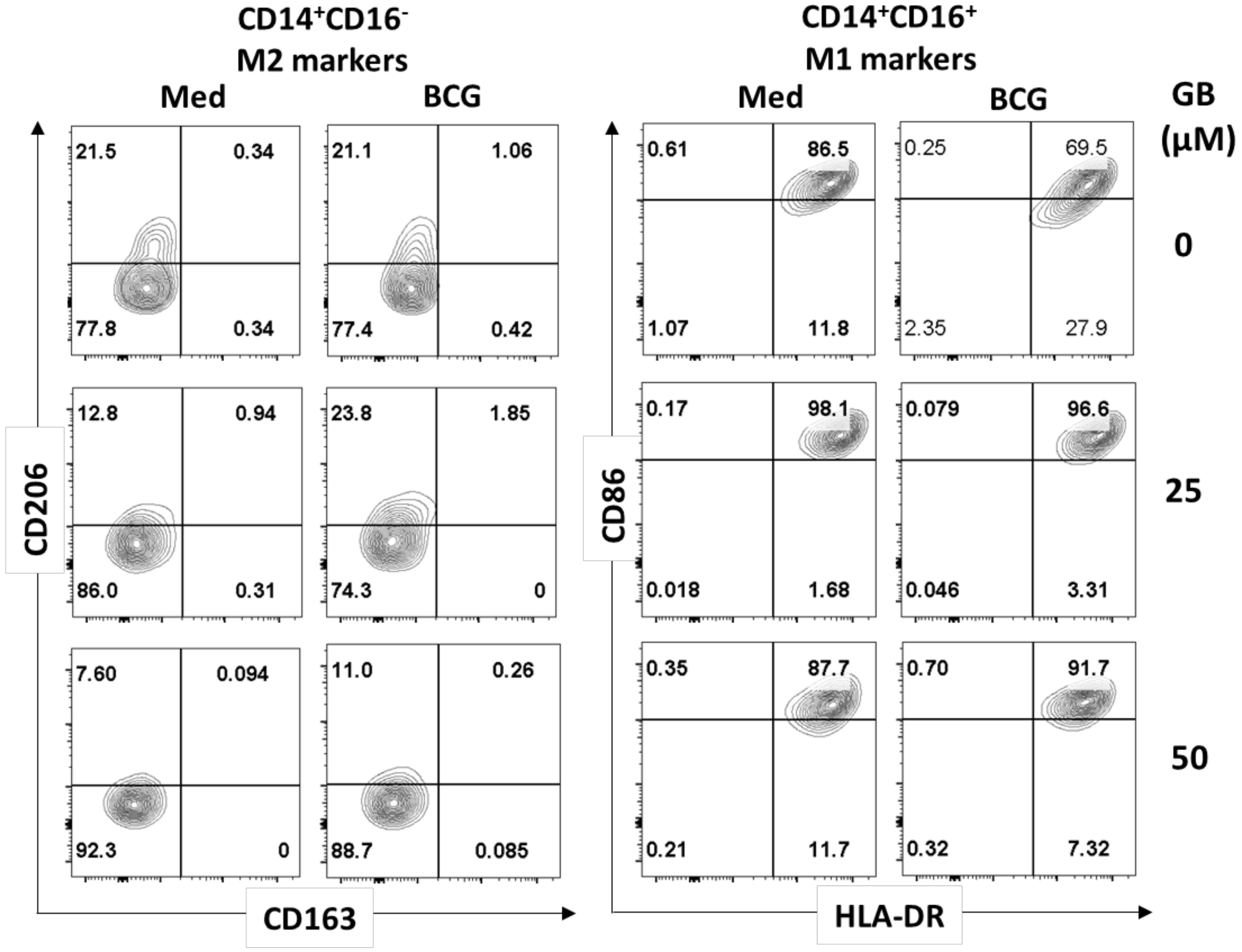

Figure S3: Effect of glibenclamide on M2 markers of $CD14^{+}CD16^{-}$ population and M1 markers of $CD14^{+}CD16^{+}$ population.

Monocytes from UK healthy individuals (n=10) were treated with glibenclamide (GB, 0, 25, 50 μM) for 30 min., incubated with 100 CFU per well of BCG or RPMI medium (Med) for 96 h and then analysed for M1 and M2 markers by flow cytometry. After exclusion of debris and doublets, monocytes were detected as $CD14^{+}CD16^{-}$ and $CD14^{+}CD16^{+}$ populations and then further analysed for M2, $CD163^{+}CD206^{+}$ and M1, HLA-$DR^{+}CD86^{+}$ expression, respectively.

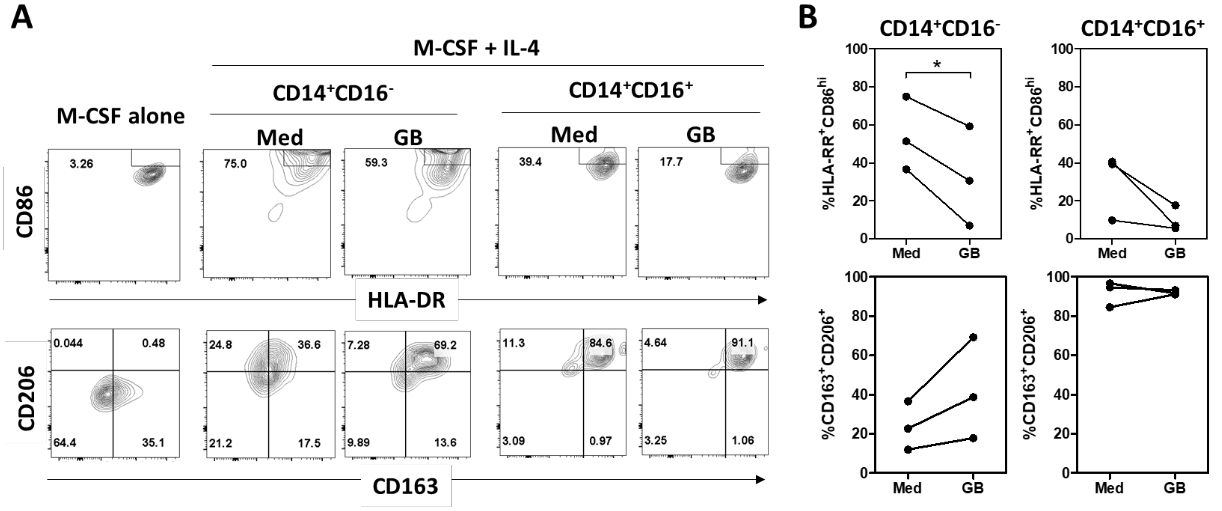

Figure S4: Glibenclamide reduces M1 markers of CD14^{+}CD16^{-} population in M2 polarized macrophages.

(A) Monocytes from UK healthy donors (n=3) were treated with 50 ng/ml monocyte-colony stimulating factor (M-CSF) for M2 differentiation. At day 5, monocyte-derived macrophages (MDM) were stimulated for 2 days with 20 ng/ml IL-4 to obtain M2 phenotypes and then analysed M1 and M2 markers by flow cytometry. (B) The percentage of M1 or M2 positive cells gated on CD14^{+}CD16^{-} and CD14^{+}CD16^{+} cells are shown. Each dot represents median with interquartile range. Statistical analysis was performed using Paired *t* test to compare between with and without GB (w/o), *P < 0.05. No asterisk, non significant.

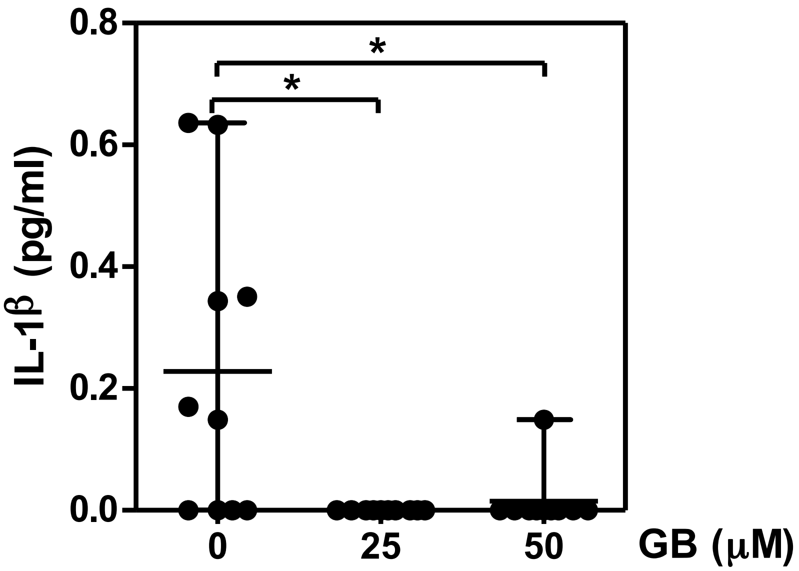

High sensitivity IL-1β ELISA: 0.125 - 8 ρg/ml

Figure S5: Glibenclamide reduces IL-1β from primary human monocytes in response to BCG.

Monocytes from UK healthy individuals were treated with glibenclamide (GB, 25, 50 μM) for 30 min. Drug-treated monocytes were incubated with 10^2 CFU per well of BCG (n=10) for 96 h and then the supernatants were collected for IL-1β detection. Error bars represent. Statistical analysis was performed using One Way ANOVA. Data are expressed as median with interquartile range. *P < 0.05. No asterisk, non significant.
